# Influence of Pavement Material and Structure on Low-Temperature Crack Resistance for Double-Layer Asphalt Surface One-Time Paving

**DOI:** 10.3390/ma18051037

**Published:** 2025-02-26

**Authors:** Bingyang Wu, Zhanchuang Han, Yunbo Mao, Shuai Wang, Hui Zhao, Shuo Zhang, Mingchen Li

**Affiliations:** 1 School of Transportation, Inner Mongolia University, Hohhot 010031, China; 0223121732@mail.imu.edu.cn; 2 Department of Civil and Environment Engineering, National University of Singapore, Singapore 117576, Singapore; 3 CCCC First Highway Engineering Co., Ltd. Fifth Engineering Co., Ltd., Beijing 100024, China; 18500829111@163.com; 4 School of Hydraulic and Civil Engineering, Ludong University, Yantai 264025, China; ws@ldu.edu.cn (S.W.); 20233410264@m.ldu.edu.cn (H.Z.); 20233410253@m.ldu.edu.cn (S.Z.); 5 School of Infrastructure Engineering, Dalian University of Technology, Dalian 116024, China; mcli@dlut.edu.cn

**Keywords:** double-layer one-time-paving pavement, paving process, thickness of structural layer, type of mixture, low-temperature crack resistance

## Abstract

The double-layer one-time-paving technology for asphalt mixtures enhances the interlayer adhesion and stability of pavement by simultaneously laying and compacting two layers of asphalt mixture, demonstrating improvements over traditional layer-by-layer paving and compaction methods. Based on this technology, the effects of paving techniques, mixture types, and structural layer thickness on the low-temperature crack resistance of pavement at −10 °C were investigated. Results indicated that, compared to traditional paving methods, the maximum tensile strain and bending strain energy density of pavement using the double-layer one-time-paving technique increased by at least 14% and 20%, respectively, under a 95% confidence level. Compared to the AC-13 + AC-25 mixture combination, the AC-16 + AC-20, AC-16 + AC-25, and AC-13 + AC-20 combinations showed increases in maximum tensile strain by at least 25%, 15%, and 15%, and in bending strain energy density by at least 57%, 38%, and 31%, respectively. Compared to the 5 cm + 5 cm thickness combination, the 4 cm + 6 cm and 3 cm + 7 cm combinations exhibited increases in maximum tensile strain by at least 14% and 22%, and in bending strain energy density by at least 16% and 29%, respectively. To effectively improve the low-temperature crack resistance of asphalt pavement at −10 °C, it is recommended to adopt a double-layer one-time-paving structure with a 3 cm AC-16 upper layer and a 7 cm AC-20 lower layer, providing insights for more durable asphalt pavements in cold climates.

## 1. Introduction

In addition to climatic factors, the pavement structure and material type have a significant impact on the performance of asphalt mixtures [1,2]. The typical asphalt pavement structure of high-grade highways in China is 4 cm AC-13 surface layer + 6 cm AC-20 middle layer + 8–12 cm AC-25 or ATB-25 (Asphalt Treated Base) or ATB-30 bottom layer, and the typical structure of trunk highways is 4 cm AC-13 surface layer + 6 cm AC-20 bottom layer or 5 cm AC-13 surface layer + 7 cm AC-20 bottom layer [3]. Limited by traditional paving technology, the construction process of each structural layer generally adopts layered paving and compaction layer by layer [4,5]. Although tack coat is sprayed between each structural layer to ensure complete bonding between layers, due to the “cold + hot” contact between the two structural layers, the mixture of the bottom layer has been fully cooled and hardened during construction and has formed a certain strength. The hot-paved mixture of the upper layer cannot be embedded into the dense and cooled bottom layer, with little aggregate interlock and weak interlayer bonding [6,7], so the interlayer bonding effect is not ideal [8]. In addition, the layered paving and rolling technology has problems such as fast heat dissipation of thin layers, long construction period, low efficiency of construction machinery [9], and high interlayer treatment cost, which affect the quality of pavement engineering and project cost [10].

The double-layer integrated paving technology of asphalt pavement is a new construction technology that completes the paving and rolling of two asphalt structural layers at one time. During construction, two layers of asphalt mixtures are paved simultaneously under high-temperature conditions, which is considered “hot + hot” contact. The asphalt in the two layers of mixtures can produce a certain bonding effect [11,12], and the particles of the two layers of asphalt mixtures can interlock with each other during the compaction process, resulting in a closer bond and interlocking between the layers. Moreover, the double-layer paving process reduces the interlayer treatment process [13], and it not only saves the use of tack coat [14], but also fundamentally solves the discontinuity of the pavement layers and enhances the interlayer bonding performance. In recent years, with the development and application of double-layer one-time-paving equipment, domestic and foreign road workers have carried out research work related to pavement materials, structures, and processes. Morgan et al. studied the application of double-layer paving technology in the construction of double-layer large-void asphalt pavements and believed that the double-layer paving technology can speed up the construction speed, adapt to adverse weather conditions, slow down the temperature loss, achieve higher compaction degree, and be more economical without spraying tack coat [15]. Gharabaghy and Chu L. et al. studied the permanent deformation resistance, fatigue performance, and shear strength of asphalt pavements constructed by double-layer paving and traditional paving, and they believed that the pavement constructed by double-layer paving has obvious advantages [16,17]. Péter Füleki believes that the interlayer interlocking is the main advantage of the double-layer paved asphalt pavement. The interlocking effect makes the asphalt layer a whole, which helps to resist the stress and deformation generated by traffic loads. At the same time, the strengthening of the interlayer bonding not only improves the high-temperature deformation resistance of the asphalt layer but also allows the use of high-quality wear-resistant and polishing aggregates [18]. In China, Wang Yanli believes that the double-layer paving technology of asphalt pavement can enhance the interlayer bonding, optimize the pavement structure, improve the compaction degree of asphalt mixtures, reduce the use of interlayer bonding materials, reduce the traffic closure time caused by construction, and reduce the impact of road construction on municipal traffic [19]. Wang Xuancang and Jiang Yingjun et al. analyzed the interlayer contact status, temperature dissipation law, and smoothness control of the double-layer-paved asphalt pavement [20,21,22,23]. Yang Yusheng et al. studied the temperature dissipation law and interlayer effect of the double-layer-paved asphalt pavement and believed that compared with the traditional construction method, the shear strength of the double-layer paving increased by 57.4%, and the effective rolling time was extended by 30–90 min [24]. Wang Lianfang compared the specimens formed by double-layer one-time paving and traditional paving methods by using the inclined shear test and believed that the shear performance of the specimens obtained by the double-layer paving method was significantly improved compared with those obtained by the traditional method [25,26,27,28,29].

Previous related studies have evaluated the interlayer bonding status, shear capacity, asphalt temperature dissipation during paving, and pavement performance based on the existing pavement structure layer thickness and mixture types, while there are few studies on the influence of structure layer thickness and mixture types on the low-temperature crack resistance of double-layer one-time-paved pavements. Therefore, this paper takes the asphalt pavement with a total thickness of 10 cm as the research object and studies the influence law of different thicknesses of the two structure layers and mixture types on the low-temperature crack resistance of the pavement, and it recommends the asphalt mixture types and pavement structure layer thicknesses based on the double-layer one-time-paving technology.

## 2. Raw Materials and Test Scheme

### 2.1. Raw Materials

#### 2.1.1. Asphalt

The 70# base asphalt was used for both the upper and lower layers, and its technical indexes are shown in Table 1. SBS (I-C) modified asphalt was used for the tack coat, and its technical requirements are shown in Table 2.

#### 2.1.2. Coarse Aggregate

The amphibolite aggregate produced by a quarry in Shangluo City, Shaanxi Province, China was used for the upper-layer coarse aggregate, and its technical indexes are shown in Table 3.

#### 2.1.3. Fine Aggregate

The limestone crushed stone produced by a company in Luonan County, Shaanxi Province, China was used for the fine aggregates of both the upper and lower layers, and its technical indexes are shown in Table 4.

#### 2.1.4. Mineral Powder

The limestone crushed stone produced by a company in Luonan County, Shaanxi Province, China was used to process the mineral powder by oneself, and its technical indexes are shown in Table 5.

### 2.2. Test Scheme

#### 2.2.1. Mixture Types and Gradations

The influence of mixture types on the low-temperature crack resistance of double-layer one-time-paved pavements was studied. AC-13 or AC-16 was proposed to be used for the surface layer, and AC-20 or AC-25 was proposed to be used for the bottom layer. The aggregate gradations of different types of mixtures are shown in Table 6. The Marshall test results of different types of mixtures at the optimal asphalt–aggregate ratio are shown in Table 7.

#### 2.2.2. Structure Layer Thickness

To investigate the influence of pavement structure layer thickness on the low-temperature crack resistance of double-layer one-time-paving pavement, the total thickness of the double-layer pavement structure was set to 10 cm, and three combinations of the thickness of the upper and lower structure layers were proposed: 3 cm for the upper layer + 7 cm for the lower layer (3 cm + 7 cm), 4 cm + 6 cm, and 5 cm + 5 cm.

#### 2.2.3. Paving Process

**Figure 1:**
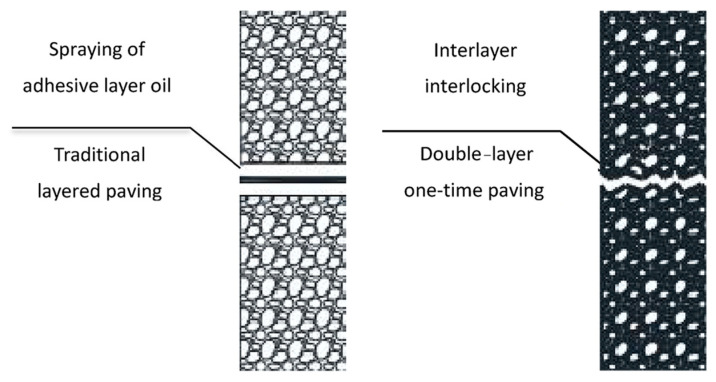
The interlayer bonding methods of different paving processes.

The impact of the layered paving and rolling process (traditional paving) and the double-layer one-time paving and rolling process (double-layer paving) of the double-layer pavement structure on the low-temperature crack resistance of the pavement was studied. Figure 1 shows the interlayer bonding methods of different paving processes. According to the test method in “Test Methods of Bitumen and Bituminous Mixtures for Highway Engineering” (JTG E20-2011) [30], low-temperature beam specimens were prepared. First, rutting plate specimens were formed indoors and then cut into prismatic beam specimens. The preparation process of indoor rutting plate specimens under different paving and rolling processes was as follows:

The size of the single-layer paving and rolling rutting plate specimen was 300 mm × 300 mm × 50 mm. The preparation process was as follows: Determine the mass of the asphalt mixture according to the density, height, and cross-sectional area of the specimen mixture. Mix the mixture, load it into the single-layer rutting plate mold of the corresponding height and tamp it thoroughly. Then, use a wheel-tracking apparatus to roll it back and forth on the surface of the mold until the surface of the specimen is rolled flat and reaches the predetermined height. Stop the rolling, and the single-layer mixture rutting plate specimen is completed.

The size of the layered paving and rolling rutting plate specimen was 300 mm × 300 mm × 100 mm. The preparation process was as follows:

(1) Preparation of the lower-layer plate: Determine the mass of the asphalt mixture according to the density, height, and cross-sectional area of the lower-layer mixture of the specimen. Mix the lower-layer mixture, load it into the single-layer rutting plate mold of the corresponding height, and tamp it thoroughly. Then, use a wheel-tracking apparatus to roll it back and forth on the surface of the mold until the thickness of the lower layer of the specimen reaches the predetermined height. Stop the rolling and the lower-layer mixture rutting plate specimen is completed.

(2) Spraying of tack coat: Demold the lower-layer mixture specimen after cooling it for at least 24 h and place it into a 300 mm × 300 mm × 100 mm double-layer rutting plate mold. Then, spray 0.45 kg/m² of tack coat and cure it for at least 2 h.

(3) Preparation of the upper-layer plate: Determine the mass of the asphalt mixture according to the density, height, and cross-sectional area of the upper-layer mixture of the specimen. Mix the upper-layer mixture, place it into the double-layer rutting plate mold with the lower-layer mixture already loaded, and tamp it thoroughly. Then, use a wheel-tracking apparatus to roll it back and forth on the surface of the mold until the total thickness of the upper and lower layers reaches 10 cm. Stop the rolling and the traditional paving rutting plate specimen is completed.

The size of the double-layer one-time paving and rolling rutting plate specimen was 300 mm × 300 mm × 100 mm, and the thickness combinations of the upper and lower structure layers were 3 cm + 7 cm, 4 cm + 6 cm, and 5 cm + 5 cm. The preparation process was as follows:

(1) Lower-layer paving: Determine the mass of the asphalt mixture according to the density, height, and cross-sectional area of the lower-layer specimen. Mix the lower-layer mixture, load it into the 300 mm × 300 mm × 100 mm double-layer rutting plate mold, and tamp it thoroughly. Perform manual pre-compaction and initial leveling, and then place it in an oven at 165 °C for heat preservation.

(2) Upper-layer paving: Determine the mass of the asphalt mixture according to the density, height, and cross-sectional area of the upper-layer specimen. Mix the upper-layer mixture, take out the double-layer rutting plate mold with the lower-layer mixture from the oven, and then load the mixed upper-layer mixture and tamp it thoroughly. Then, use a wheel-tracking apparatus to roll it back and forth on the surface of the mold until the total thickness of the upper and lower layers reaches 10 cm. Stop the rolling and the double-layer-paving rutting plate specimen is completed.

### 2.3. Low-Temperature Bending Test and Evaluation Index

According to JTG E20-2011 “Test Methods of Bitumen and Bituminous Mixtures for Highway Engineering”, a low-temperature bending test was carried out. The test temperature was −10 °C, and the loading rate was 50 mm/min. The specimen size needed to meet the requirements of length 250 mm ± 2.0 mm, width 30 mm ± 2.0 mm, and height 35 mm ± 2.0 mm. The distance between the support points was measured as 200 mm ± 0.5 mm, and the upper and lower pressure heads were kept parallel.

#### 2.3.1. Maximum Flexural Strain

The maximum flexural strain refers to the ultimate strain value at the bottom of the beam specimen when it fails. It reflects the overall deformation resistance of the mixture specimen. It is used to measure the mechanical properties of the specimen when the asphalt mixture is bent and failed at a certain temperature and loading rate. At present, it is used as an evaluation index in the low-temperature specifications of asphalt pavement construction technology in China. Generally, it is considered that the larger the maximum flexural strain, the better the low-temperature performance of the mixture. Its calculation formula is:

(1) $$\varepsilon_{B}=\frac{6hd}{L^{2}}$$

where *ε_B_* represents the maximum flexural strain, with the unit of μ*ε*; *L* represents the span of the specimen, with the unit of mm; *h* represents the height of the specimen at the fracture section, with the unit of mm; and *d* represents the mid-span deflection when the specimen fails, with the unit of mm.

#### 2.3.2. Flexural Strain Energy Density

The flexural strain energy density is proposed based on the material damage principle. Generally, it is considered that when a material fails in a low-temperature environment, it goes through three stages: crack initiation stage, increase of critical compression state, and crack formation stage. Studies have shown that the strain energy per unit volume of asphalt mixtures also follows this change law during low-temperature cracking, and it is represented by the strain energy density function of asphalt mixtures when cracking.

(2) $$\frac{dw}{dv}=\int_{0}^{\varepsilon }\sigma d\varepsilon$$

where dw/dv is the strain energy function, and *σ* and *ε* are stress and strain, respectively. The critical value of material fracture is the area under the “stress–strain” relationship curve when the stress reaches the maximum value.

According to the measured stress and strain data, it is found that they have a quadratic parabolic relationship, that is:

(3) $$\sigma =A\varepsilon^{2}+B\varepsilon +C$$

Substituting this formula into the strain energy density function, we can get:

(4) $$\frac{dw}{dv}=\int_{0}^{\varepsilon }\sigma d\varepsilon =\frac{A{\varepsilon_{0}}^{3}}{3}+\frac{B{\varepsilon_{0}}^{2}}{2}+C\varepsilon_{0}$$

where *ε*_0_ is the peak strain, and *A*, *B*, and *C* are constants.

According to the above asphalt mixture damage criterion, the larger the flexural strain energy density, the more energy is required for the material to fail, and the better the low-temperature crack resistance of the material.

## 3. Experimental Results and Analysis

### 3.1. Rutting Test Results

The test results of the low-temperature crack resistance of the double-layer pavement with different paving processes, pavement structure thicknesses, and mixture types are shown in Table 8. In the table, *ε*_B_ represents the maximum flexural strain, and W_r_ represents the flexural strain energy density.

### 3.2. Influence of Paving Process on Low-Temperature Crack Resistance of Mixture

The maximum flexural strain ε_B_ and flexural strain energy density W_r_ of the double-layer asphalt pavement under the two paving processes are shown in Table 9. In the table, ε_B__double/ε_B__traditional and W_r__double/W_r__traditional represent the ratios of the maximum flexural strain and flexural strain energy density of the double-layer paving specimen to those of the traditional paving specimen under the condition of the same mixture type and structure layer thickness.

It can be seen from Table 9 that under the condition of the same mixture type and structure layer thickness, compared with the traditional paving process, the maximum flexural strain of the double-layer paving process is increased by at least 10–29%, and the average increase is 14% under the 95% confidence level; the flexural strain energy density is increased by at least 13–34%, and the average increase is 20% under the 95% confidence level. The traditional paving process has a “cold + hot” interlayer contact, and the interlayer bonding is mainly provided by the tack coat. Aggregate interlocking cannot be formed between the layers, and it is difficult to form a skeleton structure. However, the double-layer paving process adopts a “hot + hot” interlayer contact. The interlayer interlocking effect makes the entire asphalt layer a whole, which is beneficial to the formation of the pavement structure skeleton, thus significantly improving the low-temperature crack resistance of the asphalt pavement and increasing the maximum flexural strain and flexural strain energy density of the asphalt pavement. In addition, the temperature dissipation rate of the double-layer one-time-paving asphalt mixture is lower than that of the traditional layered-paving asphalt mixture. During the paving process of the traditional layered pavement, the temperature difference between the upper and lower asphalt mixtures is large, which accelerates the heat exchange between the upper and lower layers, resulting in a rapid cooling of the upper asphalt mixture and affecting the compaction effect. The double-layer one-time-paving process involves simultaneous paving and rolling of two asphalt mixtures. This method creates a unified temperature field in the upper and lower layers, minimizing the temperature difference between them and reducing heat transfer. Additionally, the increased total thickness enhances the thermal insulation effect of the asphalt mixture. During the temperature dissipation process of the upper asphalt mixture, it can fully absorb the residual heat of the lower asphalt mixture, slow down the temperature dissipation rate, realize the “hot + hot” interlayer contact, and also extend the effective rolling time, which is beneficial to the compaction of the asphalt mixture, thus improving the low-temperature crack resistance of the asphalt pavement.

### 3.3. Influence of Mixture Type on Low-Temperature Crack Resistance of Mixture

**Figure 2:**
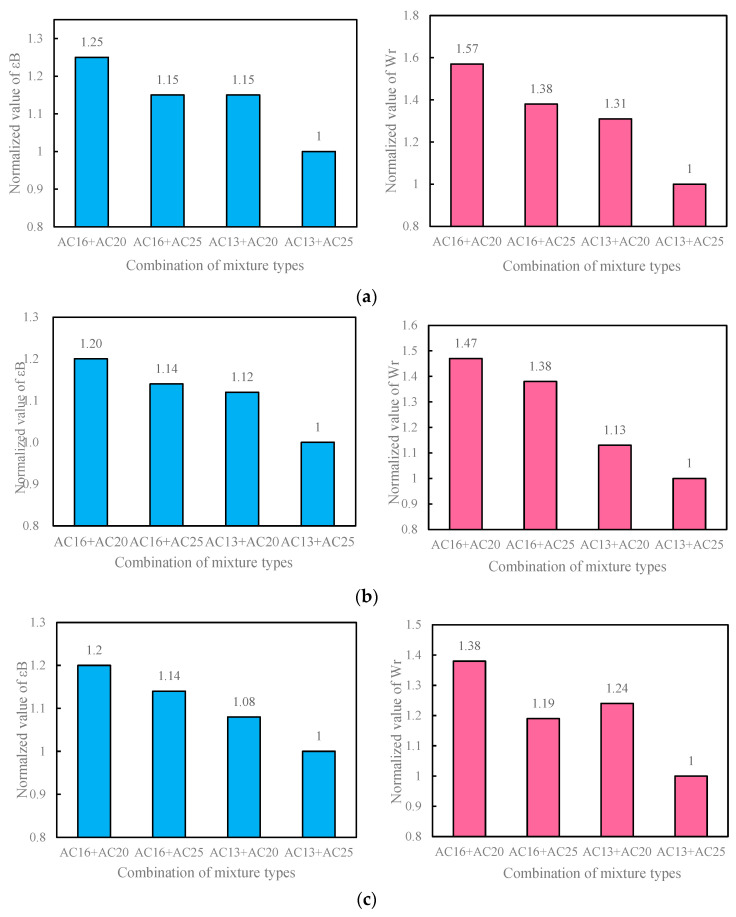
Normalized values of maximum flexural strain and flexural strain energy density under different mixture type combinations. (**a**) 3 cm + 7 cm combination; (**b**) 4 cm + 6 cm combination; (**c**) 5 cm + 5 cm combination.

The normalized values of the maximum flexural strain ε_B_ and flexural strain energy density W_r_ of the double-layer one-time-paving pavement under different mixture type combinations are compared in Figure 2.

Under the structure combination of 3 cm for the upper layer and 7 cm for the lower layer (3 cm + 7 cm), compared with the pavement with AC-13 for the upper layer and AC-25 for the lower layer (AC-13 + AC-25), the maximum flexural strains of the pavements with AC-16 + AC-20, AC-16 + AC-25, and AC-13 + AC-20 can be increased by 25%, 15%, and 15%, respectively, and the flexural strain energy densities can be increased by 57%, 38%, and 31%, respectively. Under the 4 cm + 6 cm structure layer combination, compared with the AC-13 + AC-25 pavement, the maximum flexural strains of the AC-16 + AC-20, AC-16 + AC-25, and AC-13 + AC-20 pavements can be increased by 20%, 14%, and 12%, respectively, and the flexural strain energy densities can be increased by 47%, 38%, and 13%, respectively. Under the 5 cm + 5 cm structure layer combination, compared with the AC-13 + AC-25 pavement, the maximum flexural strains of the AC-16 + AC-20, AC-16 + AC-25, and AC-13 + AC-20 pavements can be increased by 20%, 14%, and 8%, respectively, and the flexural strain energy densities can be increased by 38%, 19%, and 24%, respectively.

The test results indicate that under any structural layer combination, compared with the pavement with AC-13 in the upper layer and AC-25 in the lower layer (AC-13 + AC-25), the maximum flexural strain and flexural strain energy density of the pavements with AC-16 + AC-20, AC-16 + AC-25, and AC-13 + AC-20 are significantly increased. Among them, the improvement in the low-temperature crack resistance of the AC-16 + AC-20 pavement is the most remarkable. This is because the maximum nominal particle sizes of AC-13 and AC-25 differ greatly. AC-13 contains more fine aggregates and has a finer gradation, while AC-25 has a high content of coarse aggregates and few fine aggregates. When these two gradations are combined together, the upper and lower layers cannot be in close contact, and cannot form an interlocking connection of aggregates, which affects the formation of the strong interlocking structure of the entire road surface, resulting in a decrease in the low-temperature crack resistance performance of the road surface. When using AC-16 + AC-20 pavement, the maximum nominal particle size difference between the two gradations is not significant. When paving and compacting, the interlayer exhibits good interlocking effect, making the entire asphalt layer a whole, which is conducive to the formation of the pavement structure skeleton, and thus the low-temperature crack resistance of the asphalt pavement is significantly improved. During the pavement compaction process, the gradations with similar maximum nominal particle sizes are more likely to move and slide relative to each other, and the aggregates between the layers are more likely to approach each other until a close contact state is reached, thereby forming a dense later-stage structure. With the improvement in the integrity of the pavement structure, the load transfer level between the pavement layers is also enhanced, and thus the asphalt pavement exhibits better low-temperature crack resistance. To sum up, it is recommended that AC-16 be used for the upper layer and AC-20 for the lower layer of the double-layer one-time-paving pavement.

### 3.4. Influence of Structural Layer Thickness on Low-Temperature Crack Resistance of Mixture

**Figure 3:**
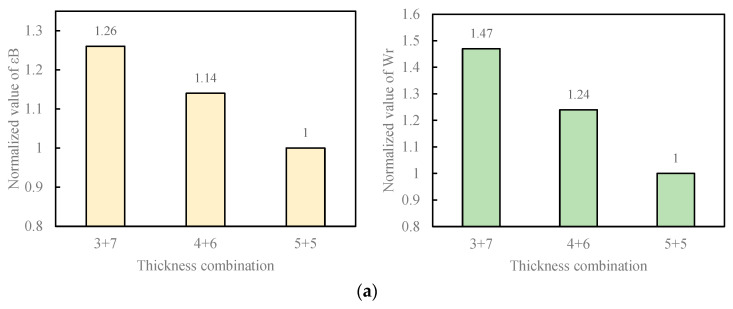

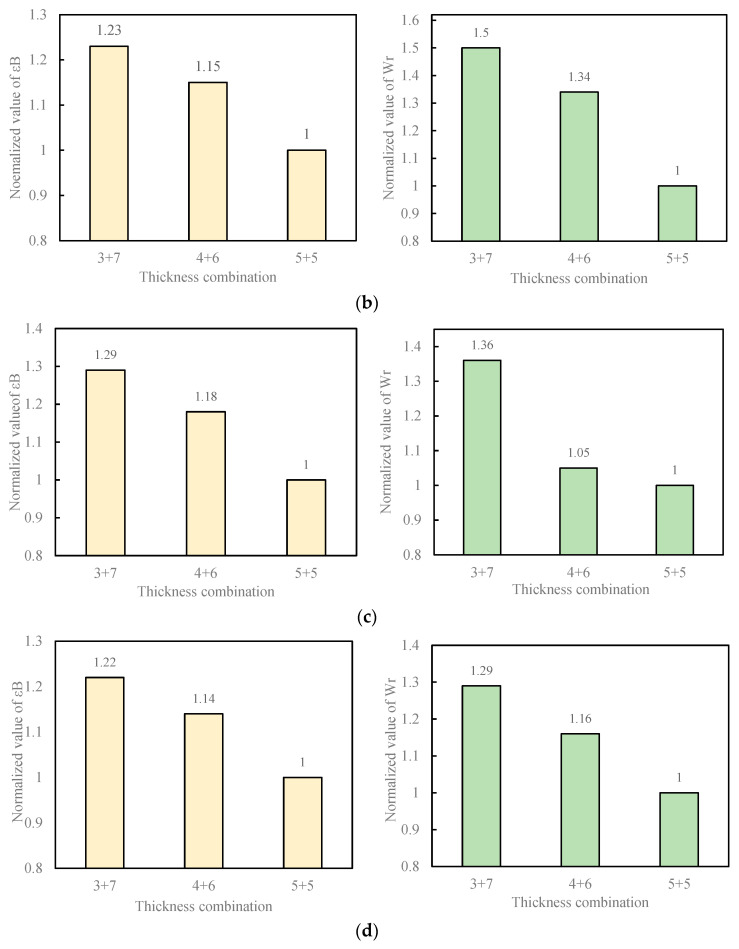
Normalized values of maximum flexural strain and flexural strain energy density under different structural layer thickness combinations. (**a**) AC-16 + AC-20 combination; (**b**) AC-16 + AC-25 combination; (**c**) AC-13 + AC-20 combination; (**d**) AC-13 + AC-25 combination.

The normalized values of the maximum flexural strain ε_B_ and flexural strain energy density W_r_ of the double-layer one-time-paving pavement under different structural layer thickness combinations are compared in Figure 3.

Under the combination of AC-16 in the upper layer and AC-20 in the lower layer (AC-16 + AC-20) mixture type, compared with the pavement with 5 cm in the upper layer and 5 cm in the lower layer (5 cm + 5 cm), the maximum flexural strains of the 3 cm + 7 cm pavement and the 4 cm + 6 cm pavement can be increased by 26% and 14%, respectively, and the flexural strain energy densities can be increased by 47% and 24%, respectively. Under the AC-16 + AC-25 mixture type combination, compared with the 5 cm + 5 cm pavement, the maximum flexural strains of the 3 cm + 7 cm pavement and the 4 cm + 6 cm pavement can be increased by 23% and 15%, respectively, and the flexural strain energy densities can be increased by 50% and 34%, respectively. Under the AC-13 + AC-20 mixture type combination, compared with the 5 cm + 5 cm pavement, the maximum flexural strains of the 3 cm + 7 cm pavement and the 4 cm + 6 cm pavement can be increased by 29% and 18%, respectively, and the flexural strain energy densities can be increased by 36% and 25%, respectively. Under the AC-13 + AC-25 mixture type combination, compared with the 5 cm + 5 cm pavement, the maximum flexural strains of the 3 cm + 7 cm pavement and the 4 cm + 6 cm pavement can be increased by 22% and 14%, respectively, and the flexural strain energy densities can be increased by 29% and 16%, respectively.

It can be seen from the above analysis of the test results that for any type of mixture, compared with the combinations of structural layer thicknesses of 5 cm + 5 cm and 4 cm + 6 cm, when the double-layer one-time-paving pavement adopts the 3 cm + 7 cm combination, the maximum flexural strain and flexural strain energy density are better, and the low-temperature crack resistance increases with the increase in the thickness of the lower structural layer. When the structural layer thickness combination changes from 5 cm + 5 cm to 4 cm + 6 cm and 3 cm + 7 cm, that is, when the thickness of the lower layer increases by 1 cm and the thickness of the upper layer decreases by 1 cm correspondingly, the maximum flexural strain and flexural strain energy density of the asphalt pavement are improved. Therefore, based on the low-temperature crack resistance of the asphalt mixture, when double-layer paving of asphalt mixture is carried out, it is recommended to adopt the “3 cm + 7 cm” thickness combination.

## 4. Engineering Application Effect

**Figure 4:**
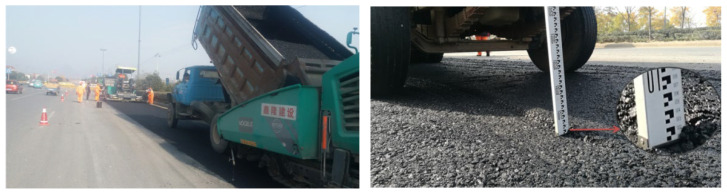
Double-layered one-time paving and testing.

The smoothness and compaction degree of the double-layer one-time-paving asphalt mixture are important factors affecting its popularization and application. In this paper, relying on the major repair project of the asphalt pavement in a certain area of Zhejiang Province, a double-layer one-time-paving asphalt pavement test road was paved to verify the application effect of the double-layer paving technology. Figure 4 shows the double-layered one-time paving and testing.

### 4.1. Compaction Degree

The compaction degree and density test results of the asphalt pavement core samples under the traditional paving process and the double-layer one-time-paving process are shown in Table 10.

It can be seen from the field core sample test results in Table 10 that the field compaction degrees of the asphalt pavements under different paving processes all meet the specification requirements (all not less than 98%). This is because after the asphalt mixture is paved on site, the paving thickness of the mixture and the on-site rolling equipment are important factors affecting the compaction degree. When the thickness of the asphalt mixture and the on-site rolling equipment are the same, the compaction degree of the pavement is positively correlated with the number of rolling passes; that is, it is closely related to the on-site effective rolling time. The effective rolling time is mainly affected by the initial paving temperature of the asphalt mixture. Therefore, the higher the initial temperature of the asphalt mixture, the easier it is to roll the pavement. When the double-layer one-time-paving process is used for rolling, due to the reduction in the temperature dissipation of the asphalt mixture during the construction process, it is easier to roll compared with the traditional rolling process. Therefore, during the rolling process, even if the number of rolling passes is appropriately reduced, it will not affect the compaction degree of the asphalt pavement.

### 4.2. Smoothness

The smoothness of the test section was detected by a laser profilometer, and the detection results of the two paving process test sections are shown in Table 11.

It can be seen from Table 11 that the smoothness of the double-layer one-time-paving and traditional layered asphalt pavements are 2.06 mm and 1.82 mm, respectively. The smoothness of the traditional layered-paving pavement is slightly higher than that of the pavement constructed by the double-layer one-time-paving process, but both meet the construction technical specifications. By analyzing the smoothness control principle of the subgrade and pavement engineering, it can be known that in the pavement structure, the smoothness of the lower-layer pavement directly affects the smoothness of the upper-layer pavement, and the smoothness has an upward mapping process and propagation law. The unsmoothness of the pavement generally first appears in the subgrade, and this unsmoothness will be continuously transmitted upward like other errors. Therefore, due to the increase in the paving thickness of the double-layer one-time-paving pavement, the smoothness will be affected to a certain extent. However, during the asphalt pavement construction process, by strictly controlling the smoothness of the subgrade and each layer above it, the smoothness of the double-layer one-time-paving pavement can still be ensured to meet the specification requirements.

## 5. Conclusions

(1) To address the cracking issues of asphalt pavement in cold regions of China, the influence of double-layer one-time-paving technology on the low-temperature crack resistance of asphalt mixtures was investigated. Compared to traditional paving methods, the maximum tensile strain of pavement using double-layer one-time paving was increased by an average of more than 14%, and the bending strain energy density was improved by an average of more than 20%. These enhancements effectively improved the durability of the pavement under low-temperature conditions.

(2) The low-temperature crack resistance of double-layer-paved pavement was significantly influenced by the types of asphalt mixtures. Compared to the AC-13 + AC-25 combination, the maximum tensile strain of the AC-16 + AC-20, AC-16 + AC-25, and AC-13 + AC-20 combinations was increased by 25%, 15%, and 15%, respectively, while the bending strain energy density was improved by 57%, 38%, and 31%, respectively. Based on the experimental results, the AC-16 + AC-20 combination was recommended as the preferred material for double-layer one-time paving.

(3) When the types of asphalt mixtures were fixed, the low-temperature crack resistance of the pavement was enhanced with the increase in the thickness of the lower layer. Compared to the 5 cm + 5 cm thickness combination, the maximum tensile strain of the 3 cm + 7 cm combination was increased by at least 22%, and the bending strain energy density was improved by at least 29%. This indicated that increasing the thickness of the lower layer could effectively improve the crack resistance of the pavement.

(4) Through laboratory tests and field engineering validation, the double-layer one-time-paving technology was recommended to effectively enhance the low-temperature crack resistance of asphalt pavement. The combination of a 3 cm AC-16 upper layer and a 7 cm AC-20 lower layer was proposed as the optimal pavement structure, demonstrating excellent crack resistance and durability in cold regions.

## Figures and Tables

**Table 1:** Singapore Esso A-70 Asphalt Technical Index.

Test Item		Measured Value	Specification/Required Value
Penetration at 25 °C (0.1 mm)		67.5	60~80
Penetration Index		−0.92	−1.5~+1.0
Ductility (5 cm/min, 15 °C, cm)		>100	≮100
Ductility (5 cm/min, 10 °C, cm)		39.2	≮25
Softening Point (°C)		49.2	≮46
60 °C Dynamic Viscosity (Pa·s)		239.4	≮180
Wax Content (%)		1.75	≯2.0
Density (15 °C, g/cm^3^)		1.015	Actual Measurement Record
Flash Point (°C)		278	≮260
Solubility (%)		99.8	≮99.5
Rotating Film Aging Test (163 °C, 85 min)	Mass Loss (%)	−0.59	≯±0.8
	Penetration Ratio (%)	62.5	≮61
	Ductility (10 °C, cm)	9.8	≮8

**Table 2:** Technical indicators of SBS (I-C) modified asphalt.

Test Item		Measured Value	Specification Value	Required Value
25 °C Density (g/cm^3^)		1.034	Actual Measurement Record	
Penetration (25 °C, 100 g, 5 s) (0.1 mm)		68	60~80	60~80
Penetration Index PI		0.56	≥−0.4	≥−0.4
Ductility (5 cm/min, 5 °C) (cm)		37	≥30	≥35
Softening Point (Ring and Ball Method) (°C)		88	≥55	≥75
135 °C Kinematic Viscosity (Pa·s)		2.4	≤3.0	1.8~3.0
Solubility (Trichloroethylene) (%)		99.7	≥99	≥99
25 °C Elastic Recovery (%)		95	≥65	≥80
Thin Film Oven Test, 163 °C, 5 h	Mass Loss (%)	−0.978	≤±1.0	≤±1.0
	Penetration Ratio (%)	73.2	≥60	≥70
	Ductility (10 °C, cm)	32.5	≥20	≥25

**Table 3:** Technical indicators of coarse aggregate.

Test Item	Technical Indexes of Aggregates of the Following Specifications				Specification/Required Value
	19~26.5 mm	9.5~19 mm	4.75~9.5 mm	2.36~4.75 mm	
Apparent Relative Density	2.861	2.848	2.826	2.811	≥2.5
Water Absorption (%)	0.34	0.39	0.68	-	≤3
Needle and Flake Particle Content (%)	10.9	8.7	8.1	-	≤15 (particle size ≥ 9.5), ≤20 (others)
Crushing Value (%)	15.9				≤26
Abrasion Value (%)	17.0				≤30
Adhesion to Asphalt (Grade)	5				≥4
Soundness (%)	4.7				≤12
Soft Stone Content (%)	1.2				≤3

**Table 4:** Technical indicators of fine aggregate.

Index	Test Result	Project Requirement Value
Sand Equivalent (%)	81.4	≥60
Methylene Blue Value (g/kg)	0.9	≤25
Angularity (s)	45.5	≥30
Apparent Density (g/cm^3^)	2.774	≥2.5
Soundness (%)	5.5	≤12

**Table 5:** Technical indicators of mineral powder.

Test Item		Measured Value	Specification/Required Value
Apparent Relative Density		2.814	≥2.5
Water Content (%)		0.2	≤1
Appearance		No Agglomeration	No Agglomeration
Particle Size Range (%)	<0.6 mm	100	100
	<0.15 mm	99.5	90~100
	<0.075 mm	92.8	75~100
Hydrophilic Coefficient		0.6	<1
Heating Stability		Good	Actual Measurement Record
CaCO_3_ Content (%)		95	≥90

**Table 6:** Different gradations of mixed materials.

Mixture Type	Percentage of Passing by Mass Through the Following Sieves (mm)												
	31.5	26.5	19	16	13.2	9.5	4.75	2.36	1.18	0.6	0.3	0.15	0.075
AC-13	-	-	-	100	90.6	81.1	55.3	39.5	28.5	14.9	9.2	8.4	7.3
AC-16	-	-	100	97.5	86.9	72.4	46.6	34.9	26.4	13.8	10.2	7.8	4.9
AC-20	-	100	94.6	84.5	75.6	65.4	44.6	34.4	22.3	16.5	9.1	7.2	5.9
AC-25	100	98.6	82.8	73.2	64.4	54.5	43.3	34.9	24.2	16.5	9.4	7.3	4.5

**Table 7:** Marshall test results at optimal oil–stone ratio.

Mixture Type	Optimal Asphalt–Aggregate Ratio (%)	Bulk Density (g/cm^3^)	Theoretical Maximum Density (g/cm^3^)	Void Ratio (%)	Saturation Degree (%)	Voids in Mineral Aggregate (%)	Stability (kN)	Flow Value (mm)
AC-13	4.8	2.525	2.605	3.1	75.1	13.9	12.6	3.8
AC-16	4.6	2.489	2.580	3.5	73.5	14.0	13.8	3.4
AC-20	4.3	2.481	2.589	4.2	72.1	14.2	14.7	2.5
AC-25	3.9	2.465	2.591	5.4	70.5	16.1	13.9	3.2

**Table 8:** Low-temperature crack resistance test results of double-layer pavement with different asphalt mixtures and pavement structure thicknesses under two paving processes.

Mixture Type		Structure Thickness (cm)		Traditional Paving		Double-Layer Paving	
Upper Layer	Lower Layer	Upper Layer	Lower Layer	ε_B_/μ_ε_	W_r_/kPa	ε_B_/μ_ε_	W_r_/kPa
AC-16	AC-20	3	7	2822	39.48	3185	46.63
		4	6	2533	33.15	2846	40.43
		5	5	2231	26.83	2498	32.45
	AC-25	3	7	2595	34.85	2931	40.11
		4	6	2417	31.03	2653	35.73
		5	5	2105	23.22	2485	26.15
AC-13	AC-20	3	7	2600	32.98	2838	38.84
		4	6	2372	25.44	2633	34.19
		5	5	2009	24.22	2588	31.27
	AC-25	3	7	2256	25.17	2504	30.52
		4	6	2119	22.54	2393	27.89
		5	5	1853	19.48	2305	25.75

**Table 9:** Maximum bending tensile strain ratio and bending strain energy density ratio with different asphalt mixtures and pavement structure thicknesses under two paving processes.

Mixture Type		Structure Thickness (cm)		ε_B__Double/ ε_B__Traditional	W_r__Double/ W_r__Traditional
Upper Layer	Lower Layer	Upper Layer	Lower Layer		
AC-16	AC-20	3	7	1.13	1.18
		4	6	1.12	1.22
		5	5	1.12	1.21
	AC-25	3	7	1.13	1.15
		4	6	1.10	1.15
		5	5	1.18	1.13
AC-13	AC-20	3	7	1.10	1.18
		4	6	1.11	1.34
		5	5	1.29	1.29
	AC-25	3	7	1.12	1.21
		4	6	1.10	1.24
		5	5	1.24	1.32

**Table 10:** Density and compaction of asphalt pavement core samples using traditional techniques and double-layer one-time-paving techniques.

Different Paving Processes	Field Density/(g/cm^3^)	Standard Density/(g/cm^3^)	Core Sample Compaction Degree/%
Double-Layer One-Time Paving	2.358	2.392	98.6
Traditional Layered Paving	2.321	2.358	98.4

**Table 11:** Traditional craftsmanship and double-layer one-time-paving technology for asphalt pavement smoothness.

Paving Process	Double-Layer One-Time Paving	Traditional Layered Paving
Smoothness/mm	2.06	1.82

## Data Availability

The original contributions presented in the study are included in the article, further inquiries can be directed to the corresponding author.

## References

[1] Ji X., Zheng N., Hou Y., Niu S. (2013). Application of asphalt mixture shear strength to evaluate pavement rutting with Accelerated Loading Facility (ALF). Constr. Build. Mater..

[2] Decky M., Hodasova K., Papanova Z., Remisova E. (2022). Sustainable adaptive cycle pavements using composite foam concrete at high altitudes in central Europe. Sustainability.

[3] Mu K. (2012). Research on Double-Layer Integrated Paving Technology of Asphalt Pavement.

[4] Mu K., Gao Z.W., Shi X., Li Y.W. (2020). Interface behavior of asphalt pavements constructed by conventional and double-decked paving methods. Materials.

[5] Fu G., Zhao Y., Ong G.P., Wang Y., Lu J. (2023). Effects of transverse cracks on the backcalculated layer properties of asphalt pavements from non-destructive testing data. J. Nondestruct. Eval..

[6] Li M., Han Z., Cheng H., Yang R., Yuan J., Jin T. (2025). Low-temperature Performance Improvement Strategies for High RAP content Recycled Asphalt Mixtures: Focus on RAP Gradation Variability and Mixing Process. Fuel.

[7] Liu J., Zhao S., Li L., Li P. (2018). Evaluation of cracking resistance in alaskan asphalt pavement with paving interlayers. J. Cold Reg. Eng..

[8] Jiang Y.J., Yi Y., Fan J.T., Tian T., Deng C.Q. (2021). Laboratory investigation on the heat dissipation regularity and road performance of different pavement structure combinations by double-layer paving. Constr. Build. Mater..

[9] Gong M.Y., Yuan M., Zhang H.T. (2024). Mechanical and functional properties of continuously paving functional asphalt mixture with double-gradation based on different volumetric ratios. J. Mater. Civ. Eng..

[10] Harvey J.T., Deacon J.A., Tsai B.W., Monismith C.L. (1995). Fatigue Performance of Asphalt Concrete Mixes and Its Relationship to Asphalt Concrete Pavement Performance in California.

[11] Gong M.Y., Zhang H.T., Wu J. (2021). CZM analysis and evaluation of influencing factors on interlayer adhesion of asphalt mixture with double-layer continuous pave. Constr. Build. Mater..

[12] Tang G.Q., Cao D.W., Zhong K., Yang X.Q. (2013). Technological study on interlayer bonding of double-layer porous asphalt pavement. Prog. Ind. Civ. Eng..

[13] Liao G.Y., Zha J.J., Lu X.Y., Wu W., Zhang W.J., Wang H., Zhang Z.S., Liu X.D. (2024). Spectral noise reduction of double-layer porous asphalt: From laboratory to field. Constr. Build. Mater..

[14] Yuan M.M., Wang J., Wang Y.Q., Shao S.G. (2021). Study on noise reduction with paving different low noise pavement materials. Appl. Sci..

[15] Morgan P.A., Stait R.E., Reeves S., Clifton M. (2007). The Feasibility of Using Twin Layer Porous Asphalt Surfaces in the UK.

[16] Gharabaghy C., Arnold P., Scharnigg K., Schulze C. (2005). State-of-the-Art Experience in the Use of the Compact Asphalt Paver for the Construction of Thin-Bed Low Noise Open-Pored 2-Course Asphalt Surfacings: State-of-the-Art Technology and Practice in the Netherlands and Germany.

[17] Chu L., Fwa T.F. (2019). Functional sustainability of single-and double-layer porous asphalt pavements. Constr. Build. Mater..

[18] Füleki P. (2009). Improving pavement performance by compact-asphalt technology. Pollack Period..

[19] Wang Y., Zhang Z., Wang B. (2007). Double-layer paving technology and performance of asphalt concrete pavement. J. China Foreign Highw..

[20] Li Y. (2011). Analysis of Economy of Heat-to-Heat Paving Technique for Asphalt Pavement. Constr. Mach. Constr. Technol..

[21] Deng C., Jiang Y., Han Z., Lin H., Fan J. (2020). Effects of paving technology, pavement materials, and structures on the fatigue property of double-layer pavements. Adv. Mater. Sci. Eng..

[22] Jiang Y., Lin H., Xue J., Han Z., Chen Z. (2020). Influences of pavement material and structure on the high-temperature stability of double-layer pavements. J. Mater. Civ. Eng..

[23] Liu M., Huang X., Xue G. (2016). Effects of double layer porous asphalt pavement of urban streets on noise reduction. Int. J. Sustain. Built Environ..

[24] Yang Y., Mu K., Wang X., Wang Z. (2012). Research on interlayer effect of double-layer paved asphalt pavement. J. Highw. Transp. Res. Dev. (Appl. Technol. Ed.).

[25] Wang L. (2010). Research on shear performance of double-layer paved pavement. Highw. Automot. Appl..

[26] Fu G., Cao D., Ong G.P., Wang J., Sha D. (2024). A viscoelastic wave propagation approach for dynamic backcalculation of layer properties of asphalt pavements under an impact load. Comput. Geotechincs.

[27] Han Z., Liu Z., Jiang Y., Wu P., Li S., Sun G., Zhang L. (2023). Engineering properties and air void characteristics of cold recycled mixtures with different compaction methods. J. Build. Eng..

[28] Han Z., Jiang D., Liu L., Sun L. (2023). Low-temperature performance improvement measures for emulsified asphalt cold recycled mixture: A comparative study. J. Mater. Civ. Eng..

[29] Xing C., Tang S., Chang Z., Han Z., Li H., Zhu B. (2024). A comprehensive review on the plant-mixed cold recycling technology of emulsified asphalt: Raw materials and factors affecting performances. Constr. Build. Mater..

[30] (2011). Test Methods of Bitumen and Bituminous Mixtures for Highway Engineering.

